# Application of cold plasma therapy for managing subclinical mastitis in cows induced by *Streptococcus agalactiae, Streptococcus uberis* and *Escherichia coli*

**DOI:** 10.1016/j.vas.2024.100378

**Published:** 2024-07-16

**Authors:** Vida Juozaitienė, Vesta Jonikė, Dalytė Mardosaitė-Busaitienė, Loreta Griciuvienė, Evelina Kaminskienė, Jana Radzijevskaja, Vilius Venskutonis, Vitas Riškevičius, Algimantas Paulauskas

**Affiliations:** Department of Biology, Faculty of Natural Sciences, Vytautas Magnus University, K. Donelaičio Str. 58, LT-44248 Kaunas, Lithuania

**Keywords:** Dairy cows, Mastitis, therapy, Cold plasma, Milk, Somatic cell count, Ruminants, Herd health

## Abstract

The primary objective of this study was to assess the effectiveness of cold plasma therapy in managing subclinical mastitis in cows caused by *Streptococcus agalactiae, Streptococcus uberis* and *Escherichia coli*. After detection of mastitis pathogens, 38 cows were selected for cold plasma therapy for five days. On the fifth day of treatment, the mastitis agents were re-examined and no causative agents were identified. An additional evaluation conducted 28 days later confirmed the absence of mastitis. Cow productivity, milk composition and quality indicators were assessed at the beginning of the experiment and 32 days from the start (28 days after treatment cessation). After the mastitis treatment, the somatic cell count decreased significantly by between 2.89 and 7.09 times, and the milk yield of the cows at the end of the experiment increased from 0.63 kg per day to 2.82 kg per day (*P* < 0.01). These results highlight the potential of this innovative approach for managing a prevalent disease that causes substantial losses in the dairy industry. Furthermore, they lay the groundwork for expanded research involving larger sample sizes.

## 1 Introduction

Mastitis in cows, which is characterised by inflammation, presents significant challenges to the health and well-being of cattle, and has substantial economic implications for farm operations (Fourichon et al., 2001; Chen et al., 2023). The condition results in reduced milk production due to inflammation-induced pain and discomfort, leading to a decline in milk yield. Additionally, the cost of treating mastitis can be high, adding to the financial burden on farmers (Wilson et al., 1997; Kulkarni & Kaliwal, 2013). Aside from the considerable economic losses associated with this ailment, mastitis has significant zoonotic potential and has been correlated with the escalating evolution and rapid emergence of multidrug-resistant strains worldwide (Pol & Ruegg, 2007; Oliver et al., 2011; Beyene et al., 2017). Thus, mastitis in milking cows can pose a threat not only to farm profitability, but also to human health and expenses related to the use of antibiotics for disease treatment and issues related to antibiotic resistance (Pascu et al., 2022). Therefore, new, effective and safe methods to treat mastitis in cows are always being sought.

Depending on the infection, mastitis can manifest in distinct forms as clinical mastitis (CM) and subclinical mastitis (SCM). Clinical mastitis typically presents with a sudden onset of redness, swelling, heat and pain in the affected milk quarter, resulting in a significant reduction in lactation. Other symptoms include the thinning and yellowing of milk, the presence of flocculent material and an elevated body temperature (Wilson et al., 1997; Kulkarni & Kaliwal, 2013). In most cases, however, the disease is predominantly marked by subclinical mastitis, for which there are no evident external signs. A key indicator of subclinical mastitis is an elevated somatic cell count in the milk (Schwarz et al., 2011). Cows surpassing 200,000 somatic cells/mL are classified as subclinically infected (Miglior et al., 2007). The array of udder pathogens is diverse, and the nature of the pathogen plays a crucial role not only in determining the manifestation of intramammary infections as acute or subclinical mastitis, but also in influencing the broader immune response. Specific pathogens can trigger distinct defence mechanisms, impacting the somatic cell count in milk, a standard parameter for assessing udder health status (Pighetti & Elliott, 2011).

Multiple research teams have explored the potential of plasma-activated water (PAW) for treating mastitis. PAW is produced by treating water with cold atmospheric plasma (CAP). Variable parameters, such as temperature, pulses, frequency, carrier gas and plasma-forming voltage, can be controlled throughout this process. Teng et al. (2022) found that PAW effectively destroyed *Staphylococcus aureus* in vitro. A study by Chiappim et al. (2021) demonstrated that PAW using a gliding arc exhibits significant inactivation potential against *S. aureus, Escherichia coli* and *Candida albicans*. According to their research, cold plasma has shown promising results in managing several bacterial diseases in veterinary medicine. Nevertheless, there is still ample room for exploration in this field (Metelmann et al, 2018; Wang et al, 2021). Currently, most cold plasma (CP) devices rely on high-voltage discharge systems, which can be intricate and expensive to operate. There is a need for the development of more compact and cost-effective plasma sources that can be seamlessly integrated into veterinary clinics. Additionally, research is required to assess the efficacy of these new plasma sources at controlling various bacterial infections.

The main objective of the study was to assess the impact of cold plasma therapy in managing subclinical mastitis in cows caused by *Streptococcus agalactiae, Streptococcus uberis* and *E. coli*. The study hypothesised that cold plasma therapy has a positive effect on the treatment of mastitis in cows, and on milk production and quality traits.

## 2 Materials and methods

### 2.1 Study design

This study was conducted on a commercial dairy farm in central Lithuania between September and late October 2023. It was conducted with the approval of the Environmental Protection Department in the Ministry of Environment (permit no. AAA 2019-04-01).

The cows were kept in the barn for the entire year, following a zero-grazing system. The total mixed feed ration (TMR) was balanced to fit the energy and nutrient requirements of a 550-650 kg Holstein cow. Based on their somatic cell count (SCC >200,000/ml), 38 cows were selected for the study. The selected cows exhibited no clinical symptoms of other diseases. A microbiology analysis of mastitis agents was performed for all the selected cows, with the treatment methods selected according to the pathogens identified. The cows were treated with plasma-activated tap water (PAW, *n* = 19) using at least 60 litres of tap water per day per affected cow (according to physiological and productivity needs) or with plasma-activated saline injections (PAS; 10 ml intramuscular injection once daily per cow, *n* = 19).

The treatments were administered for five days. On the fifth day of the experiment, cow milk samples were re-examined for mastitis agents. Cow productivity, milk composition and quality indicators were assessed at the beginning of the experiment (day 1) and 32 days from the start of the experiment (28 days after the end of treatment). At the end of the experiment, cow milk samples were analysed for mastitis for the third time.

### 2.2 Measurements

Before taking the sample, each teat was cleaned with a tissue moistened with 70 % ethanol solution, and the first three to four streams of milk were discharged. Milk from each quarter of one cow was combined into a 45 ml sample, which was then preserved using boric acid (Merck KGaA, Darmstadt, Germany). The samples were kept at 4 ± 2°C in the refrigerator for a maximum of three days. The recommendations of Harmon et al. (1999) for bacterial identification were adhered to, which included consideration of morphology, haemolysis type, gram-staining, catalase response and biochemical parameters. *S. uberis* and *S. agalactiae* were identified in part by colony morphology and esculin hydrolysis on Edward's medium. Colonies that developed on Drigalski and Chromocult® Coliform Agar and had dark blue or purple coloration were thought to be *E. coli*.

The milk sample analyses were conducted by JSC “Pieno tyrimai”, which performs tests on the milk productivity, composition and quality of controlled cows. The analysis of milk composition, including the content of fats, proteins and lactose, was performed using the infrared mid-range meter “LactoScopeFTIR” (FT1.0. 2001; Delta Instruments, Drachten, The Netherlands). The examination of somatic cell count in milk was conducted using a “Somascope” (CA-3A4, 2004, Delta Instruments, Drachten, The Netherlands) based on the principles of flow cytometry.

### 2.3 Application of cold plasma therapy for managing subclinical mastitis in cows

For the management of subclinical mastitis in the cows, two types of liquid media – plasma-activated water (PAW) and plasma-activated saline (PAS) for indirect treatment of the target (making a plasma-activated liquid and then bringing it into contact with the target) – were prepared. For the preparation of PAW and PAS, multiphase non-thermal plasma discharge was used to increase the aqueous reactions of plasma reactive species with liquid and promote the mass transfer from the gaseous phase to the liquid phase. Multi-hollow plasma discharge was initiated and maintained using metal electrodes coated with a functional coating and having a microcrystalline surface, ensuring uniform non-thermal plasma discharge (NTP) along the electrode surface.

In streamers of this kind, the temperature of electrons reaches 8-10 eV and the density of electrons can reach up to 1022 m^-3^, which means the liquid-gas interface is the place where NTP and a large proportion of reactive species are generated. Mass and thermal transfer are strengthened since most of the particles are produced close to the discharge surface (Liu, 2019). For the preparation of PAS, in-package plasma was used. In-package plasma is a unique and specific method for generating NTP and harvesting the physiochemically reactive species produced, also known as a closed, contained or sealed discharge, with no additional gas inlet either from the open atmosphere or compressed gas bottles, as is most common for NTP (Zhou et al., 2022). Plasma discharge was maintained using ambient air as the gas to create the plasma at atmospheric pressure.

For PAW preparation, the input voltage was pulsed DC with a peak voltage (VP) of 45 kV at a DC pulse frequency of 22 kHz. The discharge power was 135 W. For PAS preparation, input voltage was bipolar AC with a peak voltage (VP) of 11 kV at an AC frequency of 9.0 kHz. The discharge power was 75 W.

### 2.4 Statistical analysis of data

Statistical analysis of the study data was conducted using SPSS 25.0 software (IBM Corp., released 2017, IBM SPSS Statistics for Windows, Version 25.0, Armonk, NY: IBM Corp.). The statistical characteristics of the sample (n), including arithmetic mean (M) and standard error (SE), were calculated. Before analysis, the normality of milk trait data for all the cows was assessed using the Shapiro-Wilk normality test. The number of somatic cells in the milk (SCC) was transformed into a somatic cell score (SCS) using the formula SCS = (log^2^ (SCC/100)) + 3 (Ali, 1980) to achieve a normal distribution. The SCS data were used solely to assess the significance of the difference between the compared groups.

The comparison of identical milk parameters, along with their values at the beginning and end of the experiment, was conducted using a paired samples t-test. One-way ANOVA with post-hoc criteria was employed to evaluate differences during the same period of the experiment between lactations (two groups, least significant difference (LSD) criterion), mastitis agents (three groups, Bonferroni criterion) and treatment methods (two groups, LSD criterion).

Fisher's exact test was used as a statistical significance test in the analysis of tables of categorical variables to examine the frequency of milk samples based on the causes of mastitis and the cows’ lactation. A significance level of *P* < 0.05 was considered statistically significant for all tests.

## 3 Results

### 3.1 Distribution of cows by mastitis agent in milk samples and lactation number

On the first day of the experiment, mastitis agents were detected in a total of 38 cows. The prevalence of detected mastitis agents is shown in Table 1. Lactation numbers did not exhibit a relationship with the causative species of mastitis. The microbiological analysis of mastitis agents in milk samples from the cows showed that *S. uberis* was dominant (42.1 %) in milk samples from the primiparous cow group, while *S. agalactiae* was most prevalent (57.9 %) in the multiparous cow group.

**Table 1:** Distribution of cows by lactation number, identified mastitis agent at the beginning of the experiment, and treatment method.

Mastitis agents	Number of cows by lactation		Number of cows by treatment method and lactation	
			PAW/PAS	PAW/PAS
	Lactation 1	Lactation 2 and >	Lactation 1	Lactation 2 and >
*E. coli*	4	2	2/2	1/1
*S. uberis*	8	6	4/4	3/3
*S. agalactiae*	7	11	4/3	5/6

After a repeated examination of milk samples for mastitis, following a four-day treatment period using either PAW (*n* = 19) or PAS (*n* = 19), no mastitis causative agents were identified. Another follow-up examination after 28 days once again confirmed the absence of mastitis.

### 3.2 Milk traits of cows categorised by mastitis agents and treatment methods

The milk traits changes by mastitis agent and treatment method at the beginning and end of the experiment are given in Table 2. The averages of all milk parameters, except for milk protein, differed significantly in their values for all cows between the start (day 1) and end (day 32) of the experiment (*P* < 0.001). At the end of the experiment (day 32), there was a significant increase in daily milk yield across all groups of cows, despite the presence of identified mastitis causative agents. In the milk samples in which *E. coli, S. agalactiae* or *S. uberis* were detected, the milk yield increased significantly, with increases from 0.63 kg per day (*P* = 0.002, *E. coli* group) to 2.65 kg (*S. agalactiae* group, *P* < 0.001) and 2.82 kg (*S. uberis* group, *P* < 0.001) compared with the start of the experiment (day 1). There was also a significant increase in the milk fat content of all the above-mentioned groups of cows: from 0.09 percentage points (*P* = 0.049, *E. coli* group) to 0.16 percentage points (*S. agalactiae* group, *P* < 0.001) and 0.21 percentage points (*S. uberis* group, *P* = 0.003). At the end of the experiment (day 32), the lactose concentration had increased in all groups of cows, but a significant increase of 0.16 percentage points (*P* = 0.001) was observed only in cows where *S. agalactiae* was present in their milk samples. There were no significant changes in the milk protein content of the cows at the end of the experiment.

**Table 2:** Milk yield and content traits of cows at the start and end of the experiment, where ^ab^ in groups categorised by mastitis agent shows a statistically significant (*P* < 0.05) difference in mean values, as indicated by different letters.

Milk traits	Mastitis agents	Start of experiment (day 1)	End of experiment (day 32)	P (between the mean values at the beginning and end of the experiment)
Milk yield (kg)	*E. coli*	39.81 ± 7.066^a^	40.43 ± 6.744 ^a^	0.002
	*S. uberis*	15.60 ± 4.626^b^	18.42 ± 4.415 ^b^	<0.001
	*S. agalactiae*	24.21 ± 4.080^ab^	26.86 ± 3.894 ^ab^	0.002
Fat (%)	*E. coli*	4.29 ± 0.147 ^a^	4.38 ± 0.104 ^a^	0.049
	*S. uberis*	3.77 ± 0.096 ^b^	3.93 ± 0.068 ^b^	<0.001
	*S. agalactiae*	3.68 ± 0.085 ^b^	3.88 ± 0.060 ^b^	0.003
Protein (%)	*E. coli*	3.47 ± 0.139 ^a^	3.51 ± 0.077 ^a^	0.442
	*S. uberis*	3.43 ± 0.091 ^a^	3.33 ± 0.050 ^a^	0.156
	*S. agalactiae*	3.26 ± 0.080 ^a^	3.37 ± 0.044 ^a^	0.189
Lactose (%)	*E. coli*	4.50 ± 0.041 ^a^	4.52 ± 0.037 ^a^	0.212
	*S. uberis*	4.45 ± 0.027 ^a^	4.48 ± 0.020 ^a^	0.077
	*S. agalactiae*	4.35 ± 0.024 ^b^	4.52 ± 0.022 ^a^	0.001

When analysing the data on cow milk composition based on lactation number, significant changes were identified only in the lactose concentration of cows in whose milk samples *S. agalactiae* had been identified at the beginning of the experiment (*P* < 0.05). At the end of the experiment, the lactose concentration in the milk of these cows increased by 0.15 percentage points (4.51 ± 0.028 % in multiparous cows) and 0.18 percentage points (4.52 ± 0.020 % in primiparous cows).

The treatment method did not demonstrate a significant effect on the milk composition or quality traits of cows, except for milk somatic cell count in the *S. agalactiae* group, where at the end of the experiment, milk SCC decreased 1.34 times more in cows that had been treated with plasma-activated saline injections (*P* < 0.05).

**Fig. 1:**
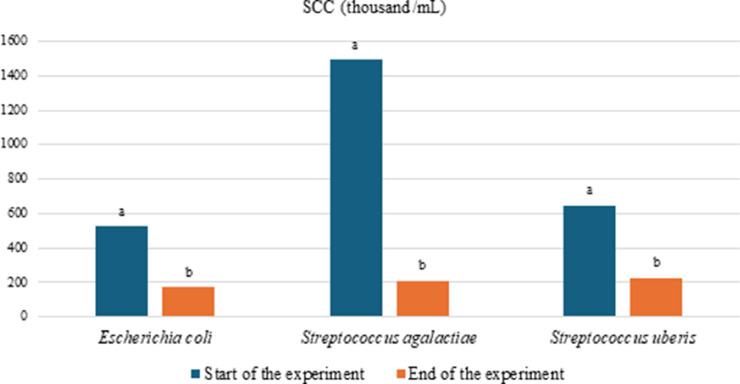
Somatic cell count in milk of cows at the start and end of experiment, where SCC is the milk somatic cell count (thousand/mL) and ^"ab"^ denotes the statistically significant difference between the mean values of SCS among groups marked with different letters: *P* < 0.05.

The somatic cell counts decreased significantly (2.89-7.09 times, *P* < 0.001) at the end of the experiment in all groups of cows based on the mastitis causative agents identified at the beginning of the experiment. The greatest reduction in somatic cells occurred in the milk of cows in whom *S. agalactiae* had been identified before treatment (Fig. 1).

### 3.4 Milk traits of cows categorised by mastitis agent and lactation number

**Fig. 2:**
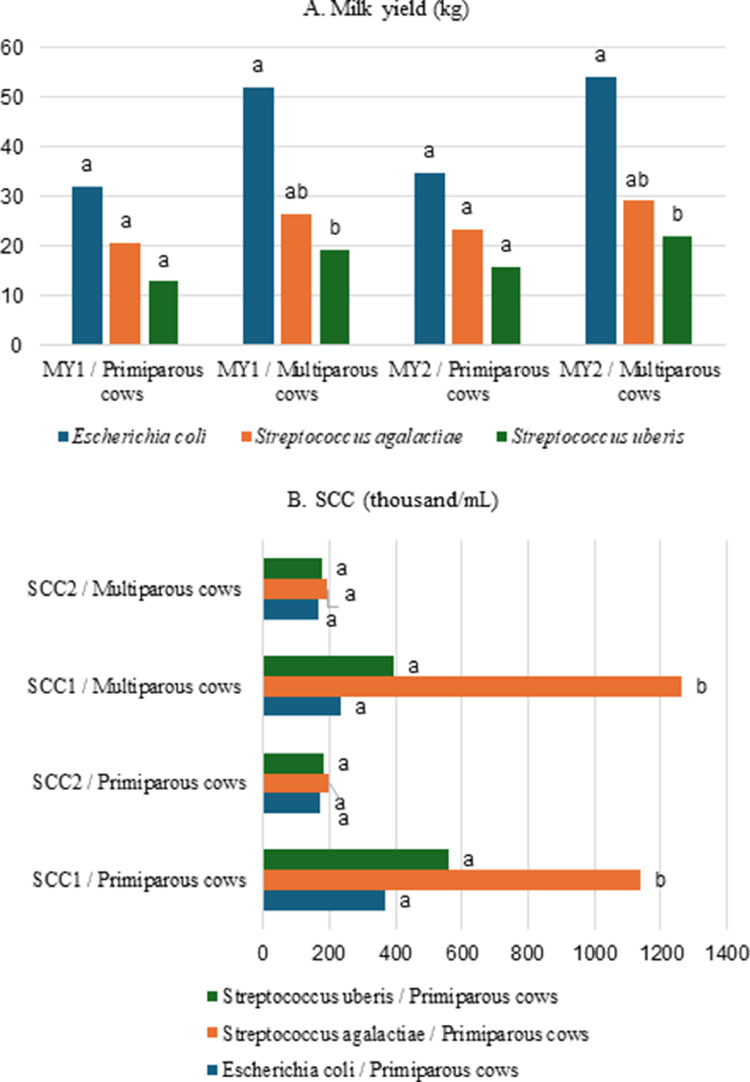
Milk yield and somatic cell count in primiparous and multiparous cows at the start and end of the experiment. MY1: milk yield at the start of the experiment, MY2: milk yield at the end of the experiment, SCC1: milk somatic cell count (thousand/mL) at the beginning of the experiment, SCC2: milk somatic cell count (thousand/mL) at the end of the experiment. ^ab^: the difference between the mean values of the groups, marked with different letters, is statistically significant: *P* < 0.05.

No statistically significant difference in milk composition was found between mastitis-causing groups based on lactation number categories. The highest milk yield was observed in all categories of cows in the *E. coli* group. The milk productivity of these multiparous cows differed significantly (2.46-2.70 times, *P* < 0.05) from the *S. uberis* group (Fig. 2 A).

The somatic cell counts at the beginning of the experiment differed significantly in both primiparous and multiparous cows with milk samples containing the identified *S. agalactiae* causative agent compared with the other groups. At the end of the experiment, no significant differences between the groups were observed in such counts (Fig. 2 B).

## 4 Discussion

The distribution of cows based on the causative agents of mastitis revealed that the most prevalent agent at the beginning of the experiment was *S. agalactiae*, classified under infectious microorganisms (47.4 %), while the least common causative agent was *E. coli* (15.8 %). *S. agalactiae* is transmitted by various means, including contaminated hands, milking containers, milking equipment, shared medication administration with the same syringe, and milking of the initial spurts of milk in the stalls. Opportunistic pathogens such as *E. coli* and *S. uberis* enter through the teat canal from the surrounding environment (Jang et al., 2017; Rana et al., 2022; Khasapane et al., 2023). *S. uberis* (42.1 %) predominated in the milk samples from primiparous cows, and *S. agalactiae* (57.9 %) in the group of multiparous cows. This indicates that the older cows could have had a weaker immune system and were more prone to being affected by infectious microorganisms.

This experimental study investigated the antimicrobial effect of PAW generated in a reactor of original construction using an atmospheric pressure surface plasma discharge and in-package plasma treatment. Plasma undergoes a series of transformations with increasing energy levels in a substance, progressing from solid to liquid, then to gas, and finally culminating in a distinct ionised water (Tabares & Junkar, 2021). Furthermore, plasma can be categorised based on the circumstances of its production either as low atmospheric or high pressure. It can also be classified into thermal or non-thermal plasmas (Murphy & Uhrlandt, 2018). Additionally, non-thermal plasma forms such as CP (cold plasma), cold atmospheric plasma (CAP) and atmospheric pressure plasma (APP) are all associated with low-temperature plasma generation, although they exhibit slight differences in terms of their properties and uses. CP denotes a partially ionised gas, with ion and electron temperatures significantly lower than those observed in traditional high-temperature plasmas (Mohseni et al., 2023).

In this study, the applied methods of cold plasma therapy for treating cow mastitis were assessed through statistical analysis, demonstrating the effectiveness of the antimicrobial activity of PAW against S. aureus, E. coli and S. uberis. It has been demonstrated that CP affects bacteria in several ways, eventually resulting in cell death (Shaw et al., 2021). According to Niedźwiedź et al. (2019), the main effects include disruption to the bacterial cell membrane, intracellular protein damage and direct DNA damage. When charged particles such as ions and electrons are present, an electric field is created that penetrates the bacterial cell wall, causing chemical connections to dissolve, membranes to breakdown and pores to form. Winter et al. (2020) also underlines how oxidative stress directly affects proteins and bacterial DNA. Significant oxidative stress is caused by reactive oxygen radicals, which can result in lipid oxygenation, protein and cytoplasm loss in cells, and oxidative DNA damage. Cell death is the ultimate result of this when the healing system is overworked.

The physicochemical characteristics of PAW were characterised under two treatment conditions: stagnant water and plasma-activated saline at 200 rpm. PAW exhibited a pH of 3.5 and an oxidation-reduction potential (ORP) of 215 mV. These parameters are associated with long-lived reactive oxygen and nitrogen species (RONS), such as ozone (O3), hydrogen peroxide (H2O2), nitrates (NO3-), and nitrites (NO2-) (Suslow, 2004; Liao et al, 2007; Lukes et al, 2008). These results suggest innovative potential for treating this prevalent cow disease, which incurs significant losses for the dairy industry. The findings also pave the way for further research with larger sample sizes.

Furthermore, the PAW and PAS treatments could be associated with enhancements in milk production, composition and quality in cows. The most significant impact on the increase in somatic cell count in milk was found in relation to *S. agalactiae*. In the milk of cows diagnosed with this mastitis agent at the onset of the experiment, 2.30-2.82 times more somatic cells (P < 0.001) were detected compared with the other groups of cows. Conversely, the most substantial decrease in somatic cell count was observed in the milk of cows with pre-identified *S. agalactiae* before treatment (7.09 times, *P* < 0.001) (Fig. 1). Zhang et al. (2013) demonstrated that PAW can be effective in treating mastitis in cows, but more research is needed to determine the optimal conditions for PAW production and use on farms. Additional research has highlighted the distinct benefits of employing PAW in the management of inflammation and the healing process. Notably, cold plasma has been observed not only to sterilise wounds, but also to stimulate the proliferation and migration of crucial skin cells – keratinocytes and fibroblasts – thus expediting the wound-healing processes (Tipa & Kroesen, 2011; Schmidt et al., 2017). The accelerated wound-healing potential of PAW has also been demonstrated in a mouse model (Wang et al., 2021). Moreover, the advantageous effects of PAW have been explored in cancer treatment, where the formation of reactive oxygen and nitrogen species in PAW has been found to play a significant role (Kaushik et al., 2019). Despite these findings, further investigation is still required into the mechanisms underlying the impact of PAW on inflammation and treatment.

No significant differences were found in other milk characteristics. Similar results have been found in other studies. Segat et al. (2015), using a spectrophotometer, noticed that CAP of 70 kV for 15 min caused mild oxidation of milk proteins, as measured by the amount of protein-bound carbonyl groups in a comparison with the control group. Meinlschmidt et al. (2016) found that the application of low-pressure cold plasma treatment did not alter the protein content of raw milk (35.3 g/L ± 0.06) compared with the control sample (34.7 g/L ± 0.17). Kim et al. (2015) also claimed that there was no significant difference in fat value after plasma treatment of a milk sample. Only Manoharan et al. (2021) have reported a significant reduction in lactose content (44.8 g/L) at 3 ml/min flow compared with the control sample (46.4 g/L). The decrease in lactose content has been linked to the interaction between -OH radicals and lactose, leading to the abstraction of a proton from the disaccharide lactose, resulting in the formation of a sugar-free radical.

After the treatments for mastitis, the milk yield of cows at the end of the experiment increased from 0.63 kg per day (*E. coli* group) to 2.82 kg per day (*S. uberis* group) (*P* < 0.01). The most substantial increase in milk fat (0.21 percentage points) was observed in the *S. uberis* group (*P* = 0.003). A significant increase was noted in the milk lactose percentage of cows whose milk samples contained *S. agalactiae* at the start of the experiment (0.16 percentage points, *P* = 0.001).

## 5 Conclusions

In this study, the applied methods of cold plasma therapy for treating cow mastitis were assessed through statistical analysis, which demonstrated the effectiveness of the antimicrobial activity of PAW against *S. aureus, E. coli* and *S. uberis*. These results suggest that cold plasma therapy has innovative potential for treating this prevalent cow disease, which leads to significant losses for the dairy industry. The findings also pave the way for further research with larger sample sizes.

## Ethical statement

The authors confirm that the ethical policies of the journal, as noted on the journal's author guidelines page, have been adhered to and the appropriate ethical review committee approval has been received. The authors confirm that they have followed EU standards for the protection of animals used for scientific purposes.

## CRediT authorship contribution statement

**Vida Juozaitienė:** Writing – original draft, Supervision, Software, Project administration, Investigation, Data curation. **Vesta Jonikė:** Writing – original draft, Validation, Investigation. **Dalytė Mardosaitė-Busaitienė:** Methodology, Investigation. **Loreta Griciuvienė:** Validation, Methodology, Investigation. **Evelina Kaminskienė:** Methodology, Investigation, Formal analysis. **Jana Radzijevskaja:** Investigation, Conceptualization. **Vilius Venskutonis:** Validation, Investigation, Formal analysis. **Vitas Riškevičius:** Validation, Investigation, Data curation. **Algimantas Paulauskas:** Writing – review & editing, Supervision, Resources, Project administration, Funding acquisition, Conceptualization.

## Declaration of competing interest

The authors declare that they have no known competing financial interests or personal relationships that could have appeared to influence the work reported in this paper.

## Data Availability

The data presented in this study are available within the article.
